# Integrin Subunit Alpha M, ITGAM Nonsynonymous SNP Is Associated with Knee Osteoarthritis among Thais: A Case-Control Study

**DOI:** 10.3390/cimb45050265

**Published:** 2023-05-09

**Authors:** Kamphon Intharanut, Plaiwan Suttanon, Oytip Nathalang

**Affiliations:** 1 Graduate Program in Biomedical Sciences, Faculty of Allied Health Sciences, Thammasat University, Pathumtani 12120, Thailand; oytipntl@hotmail.com; 2 Thammasat University Research Unit in Health, Physical Performance, Movement, and Quality of Life for Longevity Society, Department of Physical Therapy, Faculty of Allied Health Sciences, Thammasat University, Pathumtani 12120, Thailand; plaiwan.s@allied.tu.ac.th

**Keywords:** knee osteoarthritis, human neutrophil antigen, HNA-4, risk factor

## Abstract

Knee osteoarthritis (OA), which is one of the most common degenerative joint diseases, presents a multifactorial etiology, involving multiple causative factors including genetic and environmental determinants. Four human neutrophil antigen (HNA) systems can be determined using each HNA allele by single-nucleotide polymorphisms (SNPs). However, there are no data on HNA polymorphisms and knee OA in Thailand, so we investigated the association of HNA SNPs and knee OA in the Thai population. In a case-control study, detection of HNA-1, -3, -4, and -5 alleles by polymerase chain reaction with sequence-specific priming (PCR-SSP) was performed in participants with and without symptomatic knee OA. Logistic regression models were used to estimate the odds ratio (OR) and 95% confidence interval (CI) between cases and controls. Among 200 participants, 117 (58.5%) had knee OA; 83 (41.5%) did not and were included as controls in this study. An *integrin subunit alpha M* (*ITGAM*) nonsynonymous SNP, rs1143679, was markedly associated with symptomatic knee OA. The ITGAM*01*01 genotype was identified as an important increased risk factor for knee OA (adjusted OR = 5.645, 95% CI = 1.799–17.711, *p* = 0.003). These findings may contribute to our understanding of the application prospects for therapeutic approaches to knee OA.

## 1. Introduction

Osteoarthritis (OA) is the most common degenerative joint disease and can affect both cartilage and subchondral bones. OA in joints is manifested by progressive articular cartilage destruction, subchondral bone thickening, osteophyte formation, synovial inflammation, ligament and meniscus degeneration, and capsular hypertrophy [1,2,3]. The areas most frequently affected are the knees, hips, feet, ankles, distal and proximal interphalangeal joints, first carpometacarpal joint, and lower spine [4]. The main clinical presentations are pain and stiffness of the joints [5]. Pain tolerance is unique to each person and decreases with age [6], resulting in a delayed OA diagnosis among athletes and young people [5]. The diagnostic process begins with an interview and physical examination and includes a combination of radiologic imaging and laboratory testing. The main purposes of OA treatment are to relieve pain and disability and restore functionality; however, joint replacement surgery is considered for patients with severe pain and disability from large-joint OA [5].

Knee OA is a common form of arthritis among the elderly [7]. The prevalence of knee OA varies depending on the defined symptomatic or radiographic findings and characteristics of the study population, such as age and sex. There is a 10% prevalence of symptomatic knee OA in men and 18% in women among adults aged > 60 years [8]. Knee OA, as a multifactorial disease, plays a role in joint OA development, along with old age, being female, obesity, knee injury, knee alignment, occupational use (repetitive use of joints at work), bone density, muscle weakness, and joint laxity [9]. Genetic factors are among the strong determinants of this disease. Progression and severity of the disease may be influenced by the development of multiple gene factors, which interact with different alterations produced by genetic variants. Epidemiological study estimates an approximately 40% probability of inheritability in knee OA [10]. Several studies on the influence of genetics in knee OA have reported that several genes are implicated in OA pathogenesis, including *VDR*, *COL2A*, *AGC1*, *IGF-1*, *ER alpha*, and *TGF beta* [10]. In addition, a genome-wide association study (GWAS), analyzing single-nucleotide polymorphisms (SNPs), determined different knee OA susceptibility genes according to different races. In Asian cohorts, the susceptibility genes (SNP ID) *GDF5* (rs143383) [11], *DVWA* (rs11718863) [12], *HLA-DQB1* (rs7775228) [13], and *BTNL2* (rs10947262) [13] were identified as having an association with knee OA, while in Caucasian cohorts, *COG5* (rs4730250) [14], *MCF2L* (rs11842874) [15], *TP63* (rs12107036) [16], *FTO* (rs8044769) [16], *SUPT3H/RUNX2* (rs10948172) [16], and *GNL3/GLT8D1* (rs11177/rs6976) [16] were identified.

Human neutrophil antigen (HNA) consists of five systems: HNA-1, -2, -3, -4, and -5. All HNA systems can determine *HNA* alleles using SNPs, except the HNA-2 system has not yet been completely explained [17]. The details of the HNA alleles and antigens of the five HNA systems are shown in Table 1. The *Fc gamma receptor IIIb* (*FCGR3B*), *solute carrier family 44 member 2* (*SLC44A2*), *integrin subunit alpha M* (*ITGAM*), and *integrin subunit alpha L* (*ITGAL*) genes encode *HNA-1, -3, -4,* and *-5* alleles, respectively. The *HNA-1a* (FCGR3B*01) and *HNA-1b* (FCGR3B*02) encoding alleles are contributed by the SNPs c.114C>T (rs527909462) and c.194A>G (rs448740), whereas the *HNA-1c* (FCGR3B*03) allele differs from the *HNA-1b* allele at c.223C>A (rs503038). Unlike the *HNA-1a, -1b*, and *-1c* alleles, FCGR3B*null exhibits a complete lack of the entire *FCGR3B* gene. For the HNA-3 system, alleles *HNA-3a* (SLC44A2*01) and *HNA-3b* (SLC44A2*02) are caused by an SNP position at c.455G>A (rs2288094). HNA-4 and -5 comprise two alleles each; SNPs c.230G>A (rs1143679) and c.2372G>C (rs2230433) contribute to *HNA-4a* (ITGAM*01) and *HNA-4b* (ITGAM*02), and to *HNA-5a* (ITGAL*01) and *HNA-5b* (ITGAL*02) alleles, respectively, in their encoding genes, the integrin family of leukocyte cell adhesion molecules [17,18,19].

Regarding the effects of HNA polymorphisms, the granulocyte homozygous for *HNA-1b* shows a lower affinity for phagocytosis of sensitized red cells with IgG1 and IgG3 anti-Rh monoclonal antibodies and IgG1 opsonization of bacteria than the granulocyte homozygous for *HNA-1a* [20]. Additionally, a significant association was found among Thai patients with venous thromboembolism (VTE) in a study reporting a higher risk of VTE recurrence among unprovoked VTE patients, indicating homozygosity for *HNA-3a* [21]. Furthermore, a genetic variant in *ITGAM,* the *HNA-4b* polymorphism (rs1143679), has been associated with an increased risk of systemic lupus erythematosus [22]. So far, whether or not the HNA-5 polymorphism influences integrin function remains unknown. An association between several genetic factors and knee OA in specific populations has been reported, as noted previously. However, there have been no studies exploring the relationship between HNA polymorphisms and knee OA in any population. Thus, the aim of this study was to investigate the association between HNA SNPs and knee OA in a Thai population.

## 2. Materials and Methods

### 2.1. Study Design, Participants, and Periods

**Figure 1:**
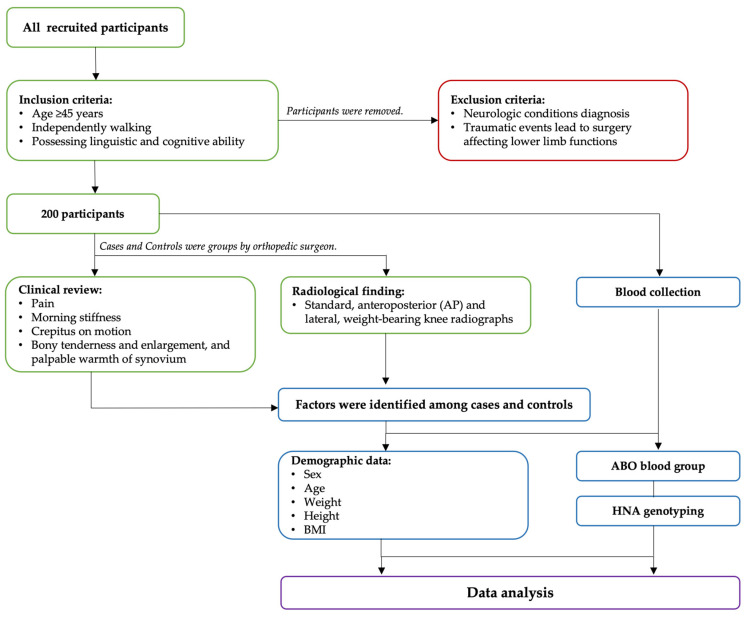
Schematic representation of workflow.

This case-control study involved individuals residing in Pathumtani Province, Thailand, and was conducted from May 2021 to March 2022. A total of 200 recruited participants met the following inclusion criteria: age 45 years or older, able to walk independently, and possessing the linguistic and cognitive ability to understand and fill out the informed consent. Participants were excluded if they were diagnosed with a neurologic condition or experienced traumatic events secondary to surgery that would affect lower limb function. Cases and controls were grouped by an orthopedic surgeon based on a review of all clinical findings (pain, morning stiffness, crepitus on motion, bony tenderness, and enlargement and palpable warmth of synovium) and radiological findings of both knees. Standard anteroposterior (AP) and lateral weight-bearing knee radiographs were performed with participants standing on both legs and were confirmed by diagnostic radiology with grades according to the Kellgren and Lawrence scale (KL; grade 0 to 4) [23]. Knee OA was diagnosed based on the presence of either clinical symptoms and physical findings along with radiographic findings, or only clinical symptoms and physical findings, while the control group had no existing medical diagnosis of knee OA. The complete workflow of the study is provided in Figure 1.

### 2.2. HNA Allele Detection by Polymerase Chain Reaction with Sequence-Specific Primer (PCR-SSP)

The individual genotyping of *HNA-1*, *-3*, *-4*, and *-5* by PCR-SSP, sequences of the primer combinations used in primer mixtures, detected alleles, SNPs, and the product size of each mixture are shown in Table 2. PCR conditions were similar to those in a related report with some modifications [19]. For each PCR reaction, the mixture consisted of 5 μL of 2× PCR reaction mixture (GoTaq^®^ Hot Start Colorless Master Mix, Promega, Madison, WI, USA), 1 μL (50–100 ng/μL) of genomic DNA, 1 μL of 5 μmol/L *HNA*-specific sense primer, 1 μL of 5 μmol/L *HNA*-specific antisense primer, and 2 μL of 2.5 μmol/L *HGH* primers (sense and antisense), running as internal control at a final volume of 10 μL. PCR was performed in a T100 thermal cycler (Bio-Rad Laboratories, Inc., Hercules, CA, USA). PCR conditions were as follows: initial denaturation at 95 °C for 10 min; 10 cycles of denaturation at 95 °C for 30 s, annealing at 64 °C for 40 s, and extension at 72 °C for 45 s; 20 cycles of denaturation at 95 °C for 30 s, annealing at 61 °C for 30 s, and extension at 72 °C for 45 s; and final extension at 72 °C for 5 min. PCR products were separated on a 1.5% agarose gel containing SYBR Safe DNA Gel Stain (Invitrogen, Paisley, UK), electrophoresed in 1× Tris borate ethylenediamine–tetraacetate (TBE) buffer at 100 volts, and visualized under a blue light transilluminator.

Regarding the *FCGR3B* deficiency and FCGR3B*null confirmation by PCR-SSP, the primers and PCR conditions were identical to those in a related report [24].

### 2.3. Statistical Analysis

Statistical analysis was performed using Microsoft Excel and SPSS version 25.0 (SPSS Inc., Chicago, IL, USA). Normally distributed continuous variables were described in terms of the mean (standard deviation, SD), and non-normal variables as the median (interquartile range, IQR). Categorical variables were summarized and expressed as absolute frequencies and percentages. As the outcome variable (knee OA) was binary, the t-test and Mann–Whitney U test were used to compare continuous variables for parametric and nonparametric tests. The multicollinearity of all explanatory variables was checked before multivariable logistic regression analysis to exclude highly interdependent variables using the variance inflation factor (VIF). Multivariable logistic regression was applied to all of the data, including continuous and categorical explanatory variables. The cut-off for the continuous predictor variable was derived by using the ROC curve for the age and BMI variables. Each HNA-1, -3, -4, and -5 genotype variable was dummy-coded into two variables representing each genotype (1 = the person possesses it, 0 = otherwise). In addition, associations were estimated by calculating the odds ratio (OR) with a 95% confidence interval (CI). Sensitivity, specificity, positive predictive value (PPV), and negative predictive value (NPV) were also calculated. Differences between KL grades were assessed using Pearson’s chi-square (χ^2^) test. A *p*-value less than 0.05 (two-tailed) was considered statistically significant.

## 3. Results

### 3.1. Description of Participants

In the 11-month recruitment period, among 200 participants we identified 117 (58.5%) as having knee OA and the remaining 83 (41.5%) as controls. Tabulated data on cases and controls by sex, age, weight, height, body mass index (BMI), and blood type are presented in Table 3. Of the 117 cases, 89 (76.1%) were women and 28 (23.9%) were men, with a median age of 62 years (IQR 53.5, 67.5). The vast majority of cases in this age group were in the 61-to-70-year-old group, comprising 39.4% of the population older than 45 years. Consequently, the proportion of women and the median age in the case group were significantly higher than those in the control group (*p* < 0.05). Median height in the case group was 155 cm (IQR 150, 162), which was significantly shorter compared to the control group (*p* = 0.001). On the other hand, no differences were found between the two groups with respect to weight, BMI, and blood type.

### 3.2. Association between HNA and Knee OA

**Figure 2:**
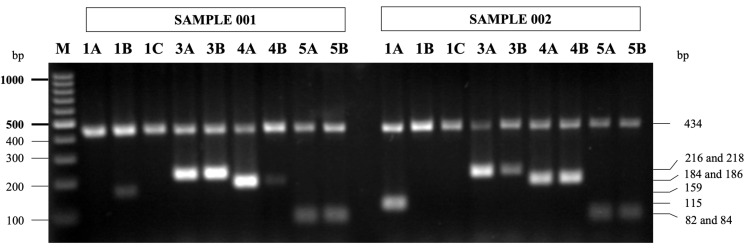
Representative gel showing *HNA* allele detection by PCR-SSP. A 434 bp amplification product of *HGH* internal control primer is present in all lanes. Allele was deduced based on presence or absence of amplification products specific for each HNA allele indicated on right border. Lane M: DNA ladder marker (GeneRuler 100 bp; Fermentas, Carlsbad, CA, USA); alleles of sample 001, lanes 1A to 5B: *HNA-1b*/*1b*, *HNA-3a*/*3b, HNA-4a*/*4b*, and *HNA-5a*/*5b*; sample 002, lanes 1A to 5B: *HNA-1a*/*1a*, *HNA-3a*/*3b, HNA-4a*/*4b*, and *HNA-5a*/*5b*.

The results of our HNA allele and genotype detection by PCR-SSP show the feasibility of distinguishing the different alleles (*HNA-1a*, *-1b*, *-1c*, *-3a*, *-3b*, *-4a*, *-4b*, *-5a*, and *-5b*) in one amplification simultaneously. DNA fragments were designed for each allelic polymorphisms based on amplicon size, as shown in Figure 2. The allele and genotype frequencies of HNA variants among cases and controls are shown in Table 4. The frequency of *HNA-1a* (114C, 194A), *HNA-3b* (455A), and *HNA-5a* (2372G) alleles in the knee OA group was higher compared to the controls (0.774 vs. 0.753, 0.312 vs. 0.289l and 0.756 vs. 0.723), although the difference was not statistically significant. Regarding the HNA-4 system, the SNP c.230G>A (rs1143679) was identified. Genotype distribution demonstrated the following distribution among cases and controls: knee OA: ITGAM*01*01 = 97.5%, ITGAM*01*02 = 4.3%, ITGAM*02*02 = 0.0%; controls: ITGAM*01*01 = 81.9%, ITGAM*01*02 = 18.5%, ITGAM*02*02 = 0.0%. In the univariate analysis, the ITGAM*01*01 genotype was associated with a risk of knee OA (OR 4.941, 95% CI 1.719–14.206, *p* = 0.003). With regard to *HNA-4* allele frequencies, *HNA-4a* allele was identified as an important increased risk factor (crude OR 4.550, 95% CI 1.620–12.780, *p* = 0.004), while the *HNA-4b* allele was identified as a reduced risk factor for knee OA (crude OR 0.220, 95% CI 0.078–0.617, *p* = 0.004). In addition, the sensitivity, specificity, PPV, and NPV of *HNA-4a* for predicting knee OA were 97.86, 9.04, 60.26, and 75.00%, respectively.

When predictor variables were evaluated for collinearity, weight, height, and BMI were found to be highly correlated (VIF = 160.12, 50.23, and 111.70, respectively). Due to the significant collinearity of these variables, only BMI was retained for subsequent data analysis due to its clinical relevance in previous studies of knee OA [8]. Multivariable logistic regression was performed to derive adjusted odds of each significant variable derived from univariable analysis. The variables of sex, age, HNA-4 genotype, and BMI of the participants were retained in the model. Additionally, HNA-1, -3, and-5 genotypes were included based on clinical adjustment and literature review. The cut-off for age was 56.5 years, with 68.3% sensitivity and 53.0% specificity; thus, an age of 57 years was chosen as the rounded cut-off for the study. Additionally, the cut-off for BMI of 25.0, with a strength of 56.63% sensitivity and 54.70% specificity, was hence chosen as the cut-off for the further analysis. We performed a series of models (see Supplemental Tables S1–S15) with different genotypes and selected the final model, as shown in Table 5. The results of multivariable analysis show that three variables had a significant relationship to knee OA (*p*-value < 0.05). The most dominant factor based on the largest adjusted OR value was ITGAM*01*01 (5.645), and the smallest was age ≥ 57 years (2.485). Women have a 3.727 times greater chance of developing knee OA than men, after controlling for other variables (Table 5).

### 3.3. Frequency of Kellgren and Lawrence (KL) Grades for Pairs of Knees

**Figure 3:**
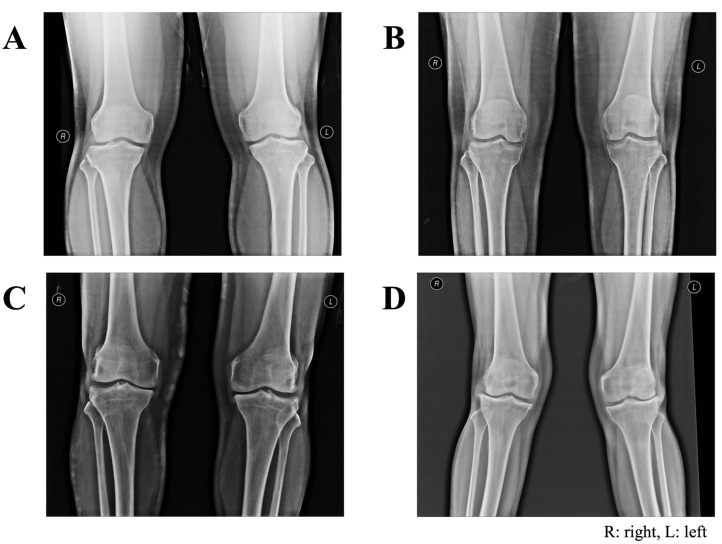
Examples of anteroposterior radiographs of paired knees presented in the current study: (**A**) bilateral KL grade 0, demonstrating normal bone density and good alignment; (**B**) bilateral KL grade 1, demonstrating possible joint space narrowing with small osteophytes in right knee; (**C**) bilateral KL grade 2, demonstrating narrow knees and patellofemoral joints with spur; (**D**) bilateral KL grade 3, demonstrating mild genu varus deformity with no displaced fracture, subchondral sclerosis with joint space narrowing, and osteophyte formation in lateral tibiofemoral joint space.

All participants underwent radiographic examination of the bilateral knees using AP and lateral views with weight-bearing. Figure 3 shows sample images of knee joints classified as KL grade 0 to 3 in this study. All 83 controls were classified as bilateral KL grade 0. Among the 117 cases of symptomatic knee OA, 115 were diagnosed with clinical and radiographic changes, and two were diagnosed as early knee OA with clinical relevance only concerning both knees (bilateral KL grade 0). Table 6 shows the frequencies of KL grades for the left and right knees in the case group. Based on the most observed radiographic changes, 58 OA knees (49.6%, 95% CI 40.2–58.9) were classified as bilateral KL grade 1. Among the others, 23 cases (19.7%, 95% CI 12.9–28.0) were classified as bilateral KL grade 2, 13 cases (11.1%, 95%CI 6.1–18.3) as unilateral KL grade 1, six cases (5.1%, 95%CI 1.9–10.8) as unilateral KL grade 2, and five cases (4.3%, 95%CI 1.4–9.7) as bilateral KL grade 3. However, unilateral or bilateral KL grade 4 was not found in this study.

The distribution of cases in each KL grade and for sex, age, and *ITGAM* genotype is summarized in Table 7. Individuals were classified by their most severe KL grade for analysis. The differences in sex, age, and *ITGAM* genotype between groups were not significant (*p* > 0.05). KL grades for women, those 57 years old or above, and those with ITGAM*01*01 genotype did not tend to be worse than the grades for men, those < 57 years old, and those with the ITGAM*01*02 genotype.

## 4. Discussion and Conclusions

The data from this study demonstrate that knee OA symptoms, including pain, stiffness, crepitus, tenderness, and enlargement and palpable warmth of the joint, were experienced by 117 participants (58.5%), whereas only 39 participants (19.5%) had knee OA (KL grade ≥ 2) observed radiographically on X-rays. Given the discrepancy between symptomatology and radiographic findings, individuals who are diagnosed with early knee OA can exhibit the symptoms without radiographic changes. No cases of X-ray findings without any symptoms were noted in this study. However, severe symptoms of knee pain have been related to advanced radiographic findings of structural pathology in different ethnic populations [25]. In addition, being female and age 57 years or above were strongly associated with the diagnosis of knee OA among these Thai participants. Women were more likely to develop higher severity than men [26]; it can be hypothesized that women may be particularly sensitive due to hormonal changes occurring across the menopausal transition [27]. The related data support the observation that women who supplemented with estrogen were 15% less likely to need total knee replacement than those who were not taking the hormone [27]. Age has been shown to be one of the strongest risk factors for OA of all joints [7,8], similar to the result of our study in which the median age of participants with knee OA was significantly higher compared to the control group. The effects of exposure to various risks factors and biologic changes of the elderly may cause more severe joint damage than would be experienced by younger adults. However, many recent studies have demonstrated that being overweight or obese, as screened by BMI, had been found to be the greatest potent risk factor for developing knee OA [28,29,30]. Such modifiable risk factors can be controlled, and their effect can be reduced by making behavioral changes.

Knee OA has a multifactorial etiology and can be considered to involve a relationship between modifiable and non-modifiable factors. Not only sex and age, but also genetic factors are among the non-modifiable risk factors that influenced knee OA. GWAS studies have revealed the correlated SNPs mentioned above, which showed a statistically significant association with knee OA in different populations [11,12,13,14,15,16], but the impact of HNA SNPs associated with this disease remains unknown. It was found that peripheral macrophages and neutrophils were found in synovium and synovium fluid, clearly demonstrating the involvement of neutrophils in the sterile inflammatory process and progression of knee OA. An α4β7 integrin plays an important role in macrophage infiltration in OA synovium [31]. However, the mechanism of neutrophil migration into OA synovium remains unclear. In the current study, the variant rs1143679 was the new locus identified as being associated with knee OA among Thai participants. We observed a directional concordance of risk between the ITGAM*01 (*HNA-4a*) allele and knee OA, while the antithetical ITGAM*02 (*HNA-4b*) allele was shown to be protective. This intergenic SNP is encoded by the *ITGAM* gene on chromosome 16p11.2 and is expressed on the CD11b/α_M_ subunit of the α_M_β_2_-integrin (CD11b/CD18, Mac-1, CR3). In terms of functionality, the β_2_ integrin is rapidly activated in leukocytes and enables adhesion of cells to counter-receptors, transmigration, phagocytosis, and oxidative burst [32,33]. *HNA-4a* and *HNA-4b* alleles differ in an ITGAM*230G>A, leading to an Arg61His substitution in the mature protein [17]. The mechanism by which changes in the polymorphism of those amino acids may lead to increased or reduced risk of knee OA remains unclear at present. It could be speculated that HNA-4 might play a role in neutrophil migration into the OA synovium and mediate OA pathogenesis. This notion warrants further experimental validation. However, strong evidence indicates the important role of integrin dysfunction in affecting OA cartilage, subchondral bone and synovium [34]. Generally, an imbalance in the anabolic and catabolic activity of the chondrocytes, especially in articular cartilage, can cause changes in the components of the extracellular matrix (ECM) in OA, as progressive factors. The integrin-mediated signaling pathway plays a key role in catabolic activity for joint destruction [35]. Likewise, excessive mechanical load signals from the ECM can trigger integrins, which work together to encourage the progression of cartilage matrix destruction in OA [36]. The interaction between integrins and cytokines secreted in the ECM, such as insulin-like growth factor and transforming growth factor beta, can also promote OA progression [37]. Changes in integrins, as mentioned above, are a key source of pathologic changes critical for the cartilage, subchondral bone, and synovium in OA [34].

Of the *ITGAM* genotypes observed in this study, the ITGAM*01*01 genotype showed the strongest correlation in knee OA cases, with a calculated OR up to 5.645 compared to the controls, as mentioned above. The ITGAM*01*02 genotype has been shown to have a statistically reduced risk of symptomatic knee OA development. The five cases were all women (mean age: 65.8 years) who received a diagnosis of pre-/early knee OA with bilateral KL grade 1. It has been acknowledged that knee OA is a multifactorial degenerative disease, so potentially the non-modifiable risk factors of sex and age could affect the diagnosis [8]. Having the ITGAM*01*02 genotype could also mean a reduced risk for knee OA and delayed disease progression. However, the occurrence of the ITGAM*02*02 genotype is very rare in Thailand and other populations [17,18,38], thus the uninformative nature of this genotyping result cannot confirm or indicate whether an individual either has a reduced risk for or is protected from developing knee OA.

Among the 117 symptomatic knee OA cases in this study, the prevalence of unilateral and bilateral KL ≥ 2 was 3.0 and 16.5%, respectively (Table 6), whereas a high prevalence was found of 12.5 and 34.1% in Beijing, and 15.2 and 19.7% in Framingham studies [38]. The high prevalence of bilateral knee OA may indicate that environmental or ethnic (genetic) factors are among the essential factors in the development of knee OA; however, we did not find an association between knee OA severity (by KL grade) and significant variables (female, ≥57 years old, and ITGAM*01*01 genotype) in this study. Among those, the knee OA severity was almost classified as an early stage of the disease (KL grades 1 and 2). From the results, the present finding of HNA SNP (rs1143679) may be closely involved in the etiology and pathogenesis of knee OA, but not in the disease activity. Moreover, severe radiographic knee OA (KL grade 4) involving large osteophytes, marked narrowing of joint space, severe sclerosis, and definite deformity of bone ends was not observed in this study. Because individuals with KL grade 4 knee OA experience great pain and discomfort when they walk or move the joints, they cannot visit research sites by themselves and were therefore excluded from this study. Among patients with KL grade 4 (advanced stage and severe OA), SNP rs1143679 should be analyzed for further study to better assess disease activity.

The major limitation of this study was the heterogenous nature of the disease in a small study cohort. The findings should be replicated in a larger sample population, in order to generalize the results observed in the current investigation and find new associations of variants, if any, in the future. In addition, further studies of the condition in different ethnic populations are needed to better characterize the risk profiles of knee OA based on genetic variants. Relatedness between individuals can lead to spurious associations between genotype and phenotype; this could not be accounted for in our model. Furthermore, participants in the case and control groups may have had different recollection regarding exposure and reverse causality, leading to a unique source of bias in case-control studies. The case-control classification should be more specific regarding the defined criteria of the dependent variable. The KL criteria are widely used because the prevalence of radiographic OA [39], defined as grade 2 or higher, is higher than that of symptomatic OA. In addition, clinical manifestations such as knee pain can be caused by other processes not related to knee OA.

In conclusion, our analysis of individual risk factors for symptomatic knee OA is the first such study in the Thai population and confirms that the SNP rs1143679 is associated with symptomatic knee OA. These results indicate that the ITGAM*01*01 genotype is a novel genetic risk factor in knee OA among Thais. These findings may contribute to understanding the beneficial application prospects for therapeutic approaches to knee OA.

## Figures and Tables

**Table 1:** HNA alleles and antigens of the five HNA systems [17].

HNA Allele		SNP	SNP ID	HNA Antigen
FCGR3B*01	*HNA-1a*	c.114C, c.194A	rs527909462, rs448740	HNA-1a
FCGR3B*02	*HNA-1b*	c.114T, c.233C	rs527909462, rs503038	HNA-1b
FCGR3B*03	*HNA-1c*	c.114T, c.233A		HNA-1c
FCGR3B*null	*HNA-1 null*	No *HNA-1* allele		HNA-1 null
*CD177*	*CD177*	No SNPs		HNA-2
SLC44A2*01	*HNA-3a*	c.455G	rs2288094	HNA-3a
SLC44A2*02	*HNA-3b*	c.455A		HNA-3b
ITGAM*01	*HNA-4a*	c.230G	rs1143679	HNA-4a
ITGAM*02	*HNA-4b*	c.230A		HNA-4b
ITGAL*01	*HNA-5a*	c.2372G	rs2230433	HNA-5a
ITGAL*02	*HNA-5b*	c.2372C		HNA-5b

FCGR3B, fc gamma receptor IIIb; HNA, human neutrophil antigen; ITGAL, integrin subunit alpha L; ITGAM, integrin subunit alpha M; SNP, single-nucleotide polymorphism; SLC44A2, solute carrier family 44 member 2.

**Table 2:** Primer sequences of reaction mixtures and product sizes used in PCR-SSP for HNA genotyping.

HNA Allele	HNA Allele	SNP	Sense Primer (5′ to 3′)	Antisense Primer (5′ to 3′)	Product Size (bp)
FCGR3B*01	*1a*	c.114C, c.194A	CAATGGTACAGGGTGCTC	CCTGGCTTGAGATGAGGT	115
FCGR3B*02	*1b*	c.114T, c.233C	CCTCAATGGTACAGCGTGCTT	ACTGTCGTTGACTGTGTCAG	159
FCGR3B*03	*1c*	c.114T, c.233A	CCTCAATGGTACAGCGTGCTT	CACTGTCGTTGACTGTGTCAT	160
SLC44A2*01	*3a*	c.455G	GACCGCTACCTCACGTACCT	CAGGGCAGTCACCATCTC	216
SLC44A2*02	*3b*	c.455A	GACCGCTACCTCACGTACCT	AGCAGGGCAGTCACCATCTT	218
ITGAM*01	*4a*	c.230G	TCATGCGAGCCCATCCG	GCCTGGGGATGTCTAGGGTAG	184
ITGAM*02	*4b*	c.230A	GCTCATGCGAGCCCATCCA	GCCTGGGGATGTCTAGGGTAG	186
ITGAL*01	*5a*	c.2372G	TTCTGATATTCCCCACCCTG	CAGTTAGACGCAGGGCTC	82
ITGAL*02	*5b*	c.2372C	TTCTGATATTCCCCACCCTG	AGCAGTTAGACGCAGGGCTG	84
*HGH*	*-*	-	CAGTGCCTTCCCAACCATTCCCTTA	ATCCACTCACGGATTTCTGTTGTGTTTC	434

BP, base pair; FCGR3B, fc gamma receptor IIIb; HGH, human growth hormone; HNA, human neutrophil antigen; ITGAL, integrin subunit alpha L; ITGAM, integrin subunit alpha M; PCR-SSP, polymerase chain reaction-sequence specific primers; SLC44A2, solute carrier family 44 member 2.

**Table 3:** Demographic data of study participants in case and control groups.

Variables	Cases (*n* = 117)	Controls (*n* = 83)	*p*-Value
Sex (F/M)	3.18/1	0.98/1	0.0001
Male, *n* (%)	28 (23.9)	42 (50.6)	
Female, *n* (%)	89 (76.1)	41 (49.4)	
Age (years): median (IQR)	62.0 (53.5, 67.5)	56.0 (49.0, 66.0)	0.007
45 to 50, *n* (%)	20 (17.1)	27 (32.5)	
51 to 60, *n* (%)	30 (25.6)	22 (26.5)	
61 to 70, *n* (%)	46 (39.4)	26 (31.4)	
>71, *n* (%)	21 (17.9)	8 (9.6)	
Weight (kg): mean (SD)	63.5 (11.8)	64.9 (12.8)	0.435
Height (cm): median (IQR)	155.0 (150.0, 162.0)	160.0 (155.0, 167.0)	0.001
BMI (kg/m^2^): median (IQR)	25.4 (22.6, 28.2)	24.5 (22.1, 27.9)	0.181
<18.5, *n* (%)	3 (2.6)	4 (4.8)	
18.5 to 24.9, *n* (%)	50 (42.6)	43 (51.9)	
25.0 to 29.9, *n* (%)	47 (40.2)	24 (28.9)	
30.0 to 34.9, *n* (%)	14 (12.0)	10 (12.0)	
35.0 to 39.9, *n* (%)	2 (1.7)	2 (2.4)	
>40.0, *n* (%)	1 (0.9)	0 (0.0)	
Blood type, *n* (%)			
A	29 (24.8)	21 (25.4)	0.934
B	40 (34.2)	24 (28.9)	0.431
O	40 (34.2)	30 (36.1)	0.775
AB	8 (6.8)	8 (9.6)	0.474

BMI, body mass index; IQR, interquartile range.

**Table 4:** Allele and genotype frequencies of HNA in cases and controls.

Genotype/Allele	Frequency, *n* (%)		Crude Odds Ratio	95% Confidence Interval		*p*-Value
	Cases, *n* = 117 (100.0)	Controls, *n* = 83 (100.0)		Lower	Upper	
HNA-1 (rs527909462, rs448740, and rs503038)						
FCGR3B*01*01	77 (65.8)	51 (61.5)	1.208	0.673	2.166	0.526
FCGR3B*01*02	27 (23.1)	23 (27.7)	0.783	0.402	1.492	0.456
FCGR3B*02*02	11 (9.3)	9 (10.8)	0.853	0.337	2.162	0.738
FCGR3B*02*03	1 (0.9)	0 (0.0)	N/A	N/A	N/A	N/A
FCGR3B*null	1 (0.9)	0 (0.0)	N/A	N/A	N/A	N/A
*HNA-1a* (114C, 194A)	181 (77.4)	125 (75.3)	1.120	0.702	1.787	0.634
*HNA-1b* (114T, 233C)	50 (21.4)	41 (24.7)	0.826	0.517	1.327	0.434
*HNA-1c* (114T, 233A)	1 (0.4)	0 (0.0)	N/A	N/A	N/A	N/A
HNA-3 (rs2288094)						
SLC44A2*01*01	54 (46.2)	40 (48.2)	0.921	0.525	1.619	0.776
SLC44A2*01*02	53 (45.3)	38 (45.8)	0.981	0.558	1.723	0.946
SLC44A2*02*02	10 (8.5)	5 (6.0)	1.458	0.480	4.434	0.507
*HNA-3a* (455G)	161 (68.8)	118 (71.1)	0.897	0.681	1.386	0.625
*HNA-3b* (455A)	73 (31.2)	48 (28.9)	1.115	0.722	1.722	0.625
HNA-4 (rs1143679)						
ITGAM*01*01	112 (97.5)	68 (81.9)	4.941	1.719	14.206	0.003
ITGAM*01*02	5 (4.3)	15 (18.1)	0.202	0.070	0.582	0.003
ITGAM*02*02	0 (0.0)	0 (0.0)	N/A	N/A	N/A	N/A
*HNA-4a* (230G)	229 (97.9)	151 (91.0)	4.550	1.620	12.780	0.004
*HNA-4b* (230A)	5 (2.1)	15 (9.0)	0.220	0.078	0.617	0.004
HNA-5 (rs2230433)						
ITGAL*01*01	72 (61.5)	50 (60.2)	1.056	0.594	1.879	0.853
ITGAL*01*02	33 (28.2)	20 (24.1)	1.238	0.650	2.357	0.517
ITGAL*02*02	12 (10.3)	13 (15.7)	0.615	0.265	1.423	0.258
*HNA-5a* (2372G)	177 (75.6)	120 (72.3)	1.190	0.757	1.871	0.450
*HNA-5b* (2372C)	57 (24.4)	46 (27.7)	0.840	0.534	1.321	0.450

FCGR3B, fc gamma receptor IIIb; HNA, human neutrophil antigen; ITGAL, integrin subunit alpha L; ITGAM, integrin subunit alpha M; N/A, not available; PCR-SSP, polymerase chain reaction-sequence specific primers; SLC44A2, solute carrier family 44 member 2. Note: The combination of other genotypes or alleles was used as the reference group to compare with genotypes or alleles of interest.

**Table 5:** Final modeling analysis of logistic regression (*n* = 200).

Variable	B	SE	Wald	Adjusted Odds Ratio	95% Confidence Interval		*p*-Value
					Lower	Upper	
Sex (female)	1.316	0.334	15.555	3.727	1.938	7.165	0.000
Age (≥57)	0.910	0.325	7.855	2.485	1.315	4.698	0.005
BMI (≥25.0)	0.448	0.318	1.986	1.565	0.839	2.919	0.159
HNA-1 genotype (FCGR3B*01*01)	0.106	0.335	0.099	1.111	0.576	2.144	0.753
HNA-3 genotype (SLC44A2*01*01)	−0.084	0.323	0.067	0.920	0.488	1.732	0.795
HNA-4 genotype (ITGAM*01*01)	1.731	0.583	8.800	5.645	1.799	17.711	0.003
HNA-5 genotype (ITGAL*01*01)	−0.149	0.340	0.193	0.862	0.443	1.676	0.661
Constant	−2.735	0.704	15.093				

B, beta coefficient; HNA, human neutrophil antigen; SE, standard error. Note: This analysis included two dummy-coded variables for knee OA outcome: sex (1 = female, 0 = male), age (1 = ≥ 57-year-old, 0 = < 57-year-old), BMI (1 = ≥ 25.0, 0 = < 25.0), HNA-1 genotype (1 = FCGR3B*01*01, 0 = otherwise), HNA-3 genotype (1 = SLC44A2*01*01, 0 = otherwise), HNA-4 genotype (1 = ITGAM*01*01, 0 = ITGAM*01*02), and HNA-5 genotype (1 = ITGAL*01*01, 0 = otherwise).

**Table 6:** Frequency of KL grades using paired knee radiographs of 117 participants with symptomatic knee OA.

KL Grades for Knee Radiographs		Number	%
Left	Right		
0	0	2	1.7
0	1	13	11.1
0	2	6	5.1
1	0	4	3.4
1	1	58	49.6
1	3	1	0.9
2	0	2	1.7
2	1	1	0.9
2	2	23	19.7
2	3	1	0.9
3	0	1	0.9
3	3	5	4.3

**Table 7:** Distribution of KL grades for 117 cases of symptomatic knee OA based on sex, age, and genotype.

Variable	Frequency, *n* (%)				χ^2^	*p*-Value
	KL0	KL1	KL2	KL3		
Sex					1.568	0.663
All	2 (1.7)	75 (64.1)	32 (27.4)	8 (6.8)		
Male	0 (0.0)	20 (17.1)	7 (6.0)	1 (0.9)		
Female	2 (1.7)	55 (47.0)	25 (21.4)	7 (6.0)		
Age					6.985	0.072
All	2 (1.7)	75 (64.1)	32 (27.4)	8 (6.8)		
<57-year-old	0 (0.0)	24 (20.5)	7 (6.0)	4 (3.4)		
≥57-year-old	2 (1.7)	51 (43.6)	25 (21.4)	4 (3.4)		
Genotype					2.925	0.403
All	2 (1.7)	75 (64.1)	32 (27.4)	8 (6.8)		
ITGAM*01*01	2 (1.7)	70 (59.8)	32 (27.4)	8 (6.8)		
ITGAM*01*02	0 (0.0)	5 (4.3)	0 (0.0)	0 (0.0)		

KL, Kellgren and Lawrence grade; ITGAM, integrin subunit alpha M; χ^2^, Pearson’s chi-square test (with 3 degrees of freedom).

## Data Availability

The datasets and materials used and/or analyzed during the current study are available from the corresponding author on reasonable request.

## Supplementary Materials

The following supporting information can be downloaded at: https://www.mdpi.com/article/10.3390/cimb45050265/s1. Tables S1–S15: The modeling analysis 1 to 15 of logistic regression.

## Abbreviations

OA	Osteoarthritis
HNA	Human neutrophil antigen
SNP	Single-nucleotide polymorphism
PCR-SSP	Polymerase chain reaction with sequence-specific priming
ITGAM	Integrin subunit alpha M
HLA	Human leucocyte antigen
FCGR3B	Fc gamma receptor IIIb
SLC44A2	Solute carrier family 44 member 2
ITGAL	Integrin subunit alpha L
AP	Anteroposterior
KL	Kellgren and Lawrence
BMI	Body mass index
ECM	Extracellular matrix

## References

[1] Chen D., Shen J., Zhao W., Wang T., Han L., Hamilton J.L., Im H.J. (2017). Osteoarthritis: Toward a comprehensive understanding of pathological mechanism. Bone Res..

[2] Loeser R.F., Goldring S.R., Scanzello C.R., Goldring M.B. (2012). Osteoarthritis: A disease of the joint as an organ. Arthritis Rheum..

[3] Martel-Pelletier J., Barr A.J., Cicuttini F.M., Conaghan P.G., Cooper C., Goldring M.B., Goldring S.R., Jones G., Teichtahl A.J., Pelletier J.P. (2016). Osteoarthritis. Nat. Rev. Dis. Primers.

[4] Simon L.S. (1999). Osteoarthritis: A review. Clin. Cornerstone.

[5] Amoako A.O., Pujalte G.G. (2014). Osteoarthritis in young, active, and athletic individuals. Clin. Med. Insights Arthritis Musculoskelet. Disord..

[6] Woodrow K.M., Friedman G.D., Siegelaub A.B., Collen M.F. (1972). Pain tolerance: Differences according to age, sex and race. Psychosom. Med..

[7] Lawrence R.C., Felson D.T., Helmick C.G., Arnold L.M., Choi H., Deyo R.A., Gabriel S., Hirsch R., Hochberg M.C., Hunder G.G. (2008). Estimates of the prevalence of arthritis and other rheumatic conditions in the United States. Part II. Arthritis Rheum..

[8] Woolf A.D., Pfleger B. (2003). Burden of major musculoskeletal conditions. Bull. World Health Organ..

[9] Zhang Y., Jordan J.M. (2010). Epidemiology of osteoarthritis. Clin. Geriatr. Med..

[10] Spector T.D., MacGregor A.J. (2004). Risk factors for osteoarthritis: Genetics. Osteoarthr. Cartil..

[11] Miyamoto Y., Mabuchi A., Shi D., Kubo T., Takatori Y., Saito S., Fujioka M., Sudo A., Uchida A., Yamamoto S. (2007). A functional polymorphism in the 5′ UTR of GDF5 is associated with susceptibility to osteoarthritis. Nat. Genet..

[12] Miyamoto Y., Shi D., Nakajima M., Ozaki K., Sudo A., Kotani A., Uchida A., Tanaka T., Fukui N., Tsunoda T. (2008). Common variants in DVWA on chromosome 3p24.3 are associated with susceptibility to knee osteoarthritis. Nat. Genet..

[13] Nakajima M., Takahashi A., Kou I., Rodriguez-Fontenla C., Gomez-Reino J.J., Furuichi T., Dai J., Sudo A., Uchida A., Fukui N. (2010). New sequence variants in HLA class II/III region associated with susceptibility to knee osteoarthritis identified by genome-wide association study. PLoS ONE.

[14] Evangelou E., Valdes A.M., Kerkhof H.J., Styrkarsdottir U., Zhu Y., Meulenbelt I., Lories R.J., Karassa F.B., Tylzanowski P., Bos S.D. (2011). Meta-analysis of genome-wide association studies confirms a susceptibility locus for knee osteoarthritis on chromosome 7q22. Ann. Rheum. Dis..

[15] Day-Williams A.G., Southam L., Panoutsopoulou K., Rayner N.W., Esko T., Estrada K., Helgadottir H.T., Hofman A., Ingvarsson T., Jonsson H. (2011). A variant in MCF2L is associated with osteoarthritis. Am. J. Hum. Genet..

[16] Zeggini E., Panoutsopoulou K., Southam L., Rayner N.W., Day-Williams A.G., Lopes M.C., Boraska V., Esko T., arcOGEN Consortium, arcOGEN Collaborators (2012). Identification of new susceptibility loci for osteoarthritis (arcOGEN): A genome-wide association study. Lancet.

[17] Flesch B.K., Curtis B.R., de Haas M., Lucas G., Sachs U.J. (2016). Update on the nomenclature of human neutrophil antigens and alleles. Transfusion.

[18] Flesch B.K., Reil A. (2018). Molecular Genetics of the Human Neutrophil Antigens. Transfus. Med. Hemother..

[19] Grabowski C., Jorks S., Kroll H. (2019). Genotyping of human neutrophil antigens 1, 3, 4 and 5 using a novel multiplex polymerase chain reaction. Transfus. Med..

[20] Bredius R.G., Fijen C.A., De Haas M., Kuijper E.J., Weening R.S., Van de Winkel J.G., Out T.A. (1994). Role of neutrophil Fc gamma RIIa (CD32) and Fc gamma RIIIb (CD16) polymorphic forms in phagocytosis of human IgG1- and IgG3-opsonized bacteria and erythrocytes. Immunology.

[21] Apipongrat D., Numbenjapon T., Prayoonwiwat W., Arnutti P., Nathalang O. (2019). Association between SLC44A2 rs2288904 polymorphism and risk of recurrent venous thromboembolism among Thai patients. Thromb. Res..

[22] Rosetti F., Mayadas T.N. (2016). The many faces of Mac-1 in autoimmune disease. Immunol Rev..

[23] Kellgren J.H., Lawrence J.S. (1957). Radiological assessment of osteo-arthrosis. Ann. Rheum. Dis..

[24] Intharanut K., Sasikarn W., Mitundee S., Nathalang O. (2019). HNA-1, -3, -4, and -5 genotyping using multiplex PCR among southern Thais: Developing continual HNA-1 null detection. J. Clin. Lab. Anal..

[25] Wang K., Kim H.A., Felson D.T., Xu L., Kim D.H., Nevitt M.C., Yoshimura N., Kawaguchi H., Lin J., Kang X. (2018). Radiographic Knee Osteoarthritis and Knee Pain: Cross-sectional study from Five Different Racial/Ethnic Populations. Sci. Rep..

[26] Srikanth V.K., Fryer J.L., Zhai G., Winzenberg T.M., Hosmer D., Jones G. (2005). A meta-analysis of sex differences prevalence, incidence and severity of osteoarthritis. Osteoarthr. Cartil..

[27] Cirillo D.J., Wallace R.B., Wu L., Yood R.A. (2006). Effect of hormone therapy on risk of hip and knee joint replacement in the Women’s Health Initiative. Arthritis Rheum..

[28] Felson D.T., Zhang Y., Anthony J.M., Naimark A., Anderson J.J. (1992). Weight loss reduces the risk for symptomatic knee osteoarthritis in women. The Framingham Study. Ann. Intern. Med..

[29] Messier S.P., Loeser R.F., Miller G.D., Morgan T.M., Rejeski W.J., Sevick M.A., Ettinger W.H., Pahor M., Williamson J.D. (2004). Exercise and dietary weight loss in overweight and obese older adults with knee osteoarthritis: The Arthritis, Diet, and Activity Promotion Trial. Arthritis Rheum..

[30] Christensen R., Bartels E.M., Astrup A., Bliddal H. (2007). Effect of weight reduction in obese patients diagnosed with knee osteoarthritis: A systematic review and meta-analysis. Ann. Rheum. Dis..

[31] Hsueh M.F., Zhang X., Wellman S.S., Bolognesi M.P., Kraus V.B. (2021). Synergistic roles of macrophages and neutrophils in osteoarthritis progression. Arthritis Rheumatol..

[32] Hynes R.O. (2002). Integrins: Bidirectional, allosteric signaling machines. Cell.

[33] Sachs U.J., Chavakis T., Fung L., Lohrenz A., Bux J., Reil A., Ruf A., Santoso S. (2004). Human alloantibody anti-Mart interferes with Mac-1-dependent leukocyte adhesion. Blood.

[34] Jin H., Jiang S., Wang R., Zhang Y., Dong J., Li Y. (2021). Mechanistic Insight Into the Roles of Integrins in Osteoarthritis. Front. Cell Dev. Biol..

[35] Song E.K., Jeon J., Jang D.G., Kim H.E., Sim H.J., Kwon K.Y., Medina-Ruiz S., Jang H.J., Lee A.R., Rho J.G. (2018). ITGBL1 modulates integrin activity to promote cartilage formation and protect against arthritis. Sci. Transl. Med..

[36] Fang T., Zhou X., Jin M., Nie J., Li X. (2021). Molecular mechanisms of mechanical load-induced osteoarthritis. Int. Orthop..

[37] Loeser R.F. (2000). Chondrocyte integrin expression and function. Biorheology.

[38] Khantisitthiporn O., Kaset C., Intharanut K., Leetrakool N., Nathalang O. (2015). Frequencies of human neutrophil antigen-4 and human neutrophil antigen-5 among Thai blood donors. Asian J. Transfus. Sci..

[39] Muraki S., Oka H., Akune T., Mabuchi A., En-yo Y., Yoshida M., Saika A., Suzuki T., Yoshida H., Ishibashi H. (2009). Prevalence of radiographic knee osteoarthritis and its association with knee pain in the elderly of Japanese population-based cohorts: The ROAD study. Osteoarthr. Cartil..

