# Supplementary material for: Integrin Subunit Alpha M, ITGAM Nonsynonymous SNP Is Associated with Knee Osteoarthritis among Thais: A Case-Control Study
Source: Curr Issues Mol Biol. 2023 May 9;45(5):4168–80. doi: 10.3390/cimb45050265 (PMC10217114; doi:10.3390/cimb45050265)
Supplement: Supplementary file 1 [file cimb-45-00265-s001.zip › cimb-2377212-supplementary.pdf]

Supplemental Table S1. The modeling analysis 1 of logistic regression with Sex, age, and BMI (n = 200).

Variable	B	SE	Wald	Adjusted odds ratio	95% Confidence interval		p-value
					Lower	Upper	
Sex (female)	1.226	0.321	14.578	**3.408**	1.816	6.395	**0.000**
Age (≥57)	0.981	0.314	9.735	**2.667**	1.440	4.938	**0.002**
BMI (≥25.0)	0.494	0.309	2.549	1.638	0.894	3.004	0.110
Constant	–1.247	0.366	11.616				

Supplemental Table S2. The modeling analysis 2 of logistic regression with Sex, age, BMI, HNA-1 genotypes (n = 200).

Variable	B	SE	Wald	Adjusted odds ratio	95% Confidence interval		p-value
					Lower	Upper	
Sex (female)	1.222	0.321	14.466	**3.395**	1.809	6.375	**0.000**
Age (≥57)	0.977	0.315	9.650	**2.657**	1.434	4.923	**0.002**
BMI (≥25.0)	0.495	0.309	2.563	1.641	0.895	3.010	0.109
HNA-1 genotype (*FCGR3B*01*01*)	0.130	0.320	0.164	1.139	0.608	2.133	0.685
Constant	–1.326	0.417	10.137				

Supplemental Table S3. The modeling analysis 3 of logistic regression with Sex, age, BMI, HNA-3 genotypes (n = 200).

Variable	B	SE	Wald	Adjusted odds ratio	95% Confidence interval		p-value
					Lower	Upper	
Sex (female)	1.227	.324	14.362	**3.412**	1.809	6.438	**0.000**
Age (≥57)	0.980	.315	9.701	**2.665**	1.438	4.939	**0.002**
BMI (≥25.0)	0.493	.309	2.544	1.638	0.893	3.004	0.111
HNA-3 genotype (*SLC44A2*01*01*)	0.010	.312	.001	1.010	0.548	1.861	0.975
Constant	–1.252	.400	9.802				

Supplemental Table S4. The modeling analysis 4 of logistic regression with Sex, age, BMI, HNA-4 genotypes (n = 200).

Variable	B	SE	Wald	Adjusted odds ratio	95% Confidence interval		p-value
					Lower	Upper	
Sex (female)	1.318	0.330	15.940	**3.738**	1.957	7.140	**0.000**
Age (≥57)	0.924	0.323	8.207	**2.519**	1.339	4.741	**0.004**
BMI (≥25.0)	0.444	0.317	1.953	1.558	0.837	2.903	0.162
HNA-4 genotype (*ITGAM*01*01*)	1.682	0.572	8.650	**5.379**	1.753	16.504	**0.003**
Constant	–2.764	0.660	17.509				

Supplemental Table S5. The modeling analysis 5 of logistic regression with Sex, age, BMI, HNA-5 genotypes (n = 200).

Variable	B	SE	Wald	Adjusted odds ratio	95% Confidence interval		p-value
					Lower	Upper	
Sex (female)	1.222	0.322	14.444	**3.394**	1.807	6.375	**0.000**
Age (≥57)	0.989	0.316	9.772	**2.687**	1.446	4.994	**0.002**
BMI (≥25.0)	0.493	0.309	2.538	1.637	0.893	3.002	0.111
HNA-5 genotype (*ITGAL*01*01*)	0.077	0.319	0.058	1.080	0.578	2.017	0.810
Constant	–1.296	0.419	9.564				

Supplemental Table S6. The modeling analysis 6 of logistic regression with Sex, age, BMI, HNA-1, and HNA-3 genotypes (n = 200).

Variable	B	SE	Wald	Adjusted odds ratio	95% Confidence interval		p-value
					Lower	Upper	
Sex (female)	1.222	0.324	14.210	**3.395**	1.798	6.410	**0.000**
Age (≥57)	0.977	0.315	9.630	**2.657**	1.433	4.926	**0.002**
BMI (≥25.0)	0.495	0.310	2.561	1.641	0.895	3.011	0.110
HNA-1 genotype (*FCGR3B*01*01*)	0.130	0.321	0.163	1.139	0.606	2.138	0.686
HNA-3 genotype (*SLC44A2*01*01*)	0.000	0.313	0.000	0.999	0.541	1.846	0.998
Constant	–1.326	0.441	9.024				

Supplemental Table S7. The modeling analysis 7 of logistic regression with Sex, age, BMI, HNA-1, and HNA-4 genotypes (n = 200).

Variable	B	SE	Wald	Adjusted odds ratio	95% Confidence interval		p-value
					Lower	Upper	
Sex (female)	1.316	0.330	15.869	**3.730**	1.952	7.129	**0.000**
Age (≥57)	0.922	0.323	8.162	**2.514**	1.336	4.733	**0.004**
BMI (≥25.0)	0.445	0.318	1.961	1.560	0.837	2.907	0.161
HNA-1 genotype (*FCGR3B*01*01*)	0.079	0.330	0.056	1.082	0.566	2.067	0.812
HNA-4 genotype (*ITGAM*01*01*)	1.678	0.573	8.575	**5.352**	1.741	16.450	**0.003**
Constant	–2.808	0.688	16.666				

Supplemental Table S8. The modeling analysis 8 of logistic regression with Sex, age, BMI, HNA-1, and HNA-5 genotypes (n = 200).

Variable	B	SE	Wald	Adjusted odds ratio	95% Confidence interval		p-value
					Lower	Upper	
Sex (female)	1.219	0.322	14.360	**3.385**	1.802	6.361	**0.000**
Age (≥57)	0.983	0.317	9.654	**2.674**	1.438	4.972	**0.002**
BMI (≥25.0)	0.495	0.310	2.553	1.640	0.894	3.008	0.110
HNA-1 genotype (*FCGR3B*01*01*)	0.121	0.323	0.141	1.129	0.599	2.128	0.708
HNA-5 genotype (*ITGAL*01*01*)	0.060	0.322	0.035	1.062	0.565	1.995	0.852
Constant	–1.359	0.452	9.022				

Supplemental Table S9. The modeling analysis 9 of logistic regression with Sex, age, BMI, HNA-3, and HNA-4 genotypes (n = 200).

Variable	B	SE	Wald	Adjusted odds ratio	95% Confidence interval		p-value
					Lower	Upper	
Sex (female)	1.310	0.332	15.532	**3.707**	1.932	7.112	**0.000**
Age (≥57)	0.928	0.323	8.244	**2.528**	1.342	4.763	**0.004**
BMI (≥25.0)	0.445	0.318	1.967	1.561	0.838	2.909	0.161
HNA-3 genotype (*SLC44A2*01*01*)	–0.066	0.321	0.042	0.937	0.500	1.756	0.838
HNA-4 genotype (*ITGAM*01*01*)	1.690	0.573	8.698	**5.417**	1.762	16.649	**0.003**
Constant	–2.736	0.673	16.528				

Supplemental Table S10. The modeling analysis 10 of logistic regression with Sex, age, BMI, HNA-3, and HNA-5 genotypes (n = 200).

Variable	B	SE	Wald	Adjusted odds ratio	95% Confidence interval		p-value
					Lower	Upper	
Sex (female)	1.224	0.324	14.252	**3.400**	1.801	6.419	**0.000**
Age (≥57)	0.988	0.317	9.741	**2.686**	1.444	4.994	**0.002**
BMI (≥25.0)	0.492	0.309	2.532	1.636	0.892	3.001	0.112
HNA-3 genotype (*SLC44A2*01*01*)	0.014	0.312	0.002	1.014	0.550	1.870	0.964
HNA-5 genotype (*ITGAL*01*01*)	0.077	0.319	0.059	1.081	0.578	2.020	0.808
Constant	–1.303	0.453	8.279				

Supplemental Table S11. The modeling analysis 11 of logistic regression with Sex, age, BMI, HNA-4, and HNA-5 genotypes (n = 200).

Variable	B	SE	Wald	Adjusted odds ratio	95% Confidence interval		p-value
					Lower	Upper	
Sex (female)	1.327	0.331	16.060	**3.771**	1.970	7.216	**0.000**
Age (≥57)	0.911	0.324	7.892	**2.486**	1.317	4.694	**0.005**
BMI (≥25.0)	0.444	0.318	1.956	1.559	0.837	2.905	0.162
HNA-4 genotype (*ITGAM*01*01*)	1.722	0.582	8.765	**5.597**	1.790	17.502	**0.003**
HNA-5 genotype (*ITGAL*01*01*)	–0.129	0.335	0.148	0.879	0.456	1.696	0.701
Constant	–2.718	0.670	16.467				

Supplemental Table S12. The modeling analysis 12 of logistic regression with Sex, age, BMI, HNA-1, HNA-3, and HNA-4 genotypes (n = 200).

Variable	B	SE	Wald	Adjusted odds ratio	95% Confidence interval		p-value
					Lower	Upper	
Sex (female)	1.307	0.333	15.426	**3.696**	1.925	7.095	**0.000**
Age (≥57)	0.926	0.323	8.204	**2.524**	1.339	4.755	**0.004**
BMI (≥25.0)	0.447	0.318	1.977	1.563	0.839	2.914	0.160
HNA-1 genotype (*FCGR3B*01*01*)	0.085	0.332	0.065	1.089	0.568	2.085	0.798
HNA-3 genotype (*SLC44A2*01*01*)	–0.073	0.322	0.051	0.930	0.495	1.748	0.822
HNA-4 genotype (*ITGAM*01*01*)	1.685	0.574	8.629	**5.392**	1.752	16.597	**0.003**
Constant	–2.781	0.697	15.915				

Supplemental Table S13. The modeling analysis 13 of logistic regression with Sex, age, BMI, HNA-1, HNA-3, and HNA-5 genotypes (n = 200).

Variable	B	SE	Wald	Adjusted odds ratio	95% Confidence interval		p-value
					Lower	Upper	
Sex (female)	1.220	0.325	14.129	**3.387**	1.793	6.398	**0.000**
Age (≥57)	0.983	0.317	9.636	**2.673**	1.437	4.974	**0.002**
BMI (≥25.0)	0.494	0.310	2.549	1.640	0.894	3.009	0.110
HNA-1 genotype (*FCGR3B*01*01*)	0.121	0.325	0.139	1.129	0.597	2.134	0.709
HNA-3 genotype (*SLC44A2*01*01*)	0.003	0.314	0.000	1.003	0.542	1.855	0.992
HNA-5 genotype (*ITGAL*01*01*)	0.060	0.323	0.035	1.062	0.564	1.998	0.852
Constant	–1.361	0.479	8.063				

Supplemental Table S14. The modeling analysis 14 of logistic regression with Sex, age, BMI, HNA-1, HNA-4, and HNA-5 genotypes (n = 200).

Variable	B	SE	Wald	Adjusted odds ratio	95% Confidence interval		p-value
					Lower	Upper	
Sex (female)	1.326	0.331	16.003	**3.765**	1.966	7.210	**0.000**
Age (≥57)	0.907	0.325	7.810	**2.477**	1.311	4.678	**0.005**
BMI (≥25.0)	0.446	0.318	1.968	1.562	0.838	2.911	0.161
HNA-1 genotype (*FCGR3B*01*01*)	0.097	0.334	0.085	1.102	0.573	2.121	0.770
HNA-4 genotype (*ITGAM*01*01*)	1.720	0.582	8.729	**5.585**	1.784	17.480	**0.003**
HNA-5 genotype (*ITGAL*01*01*)	–0.142	0.338	0.176	0.868	0.447	1.684	0.675
Constant	–2.768	0.693	15.943				

Supplemental Table S15. The modeling analysis 15 of logistic regression with Sex, age, BMI, HNA-3, HNA-4, and HNA-5 genotypes (n = 200).

Variable	B	SE	Wald	Adjusted odds ratio	95% Confidence interval		p-value
					Lower	Upper	
Sex (female)	1.318	0.333	15.652	**3.737**	1.945	7.180	**0.000**
Age (≥57)	0.914	0.325	7.932	**2.495**	1.320	4.713	**0.005**
BMI (≥25.0)	0.446	0.318	1.971	1.562	0.838	2.912	0.160
HNA-3 genotype (*SLC44A2*01*01*)	–0.074	0.322	0.053	0.929	0.494	1.744	0.818
HNA-4 genotype (*ITGAM*01*01*)	1.732	0.583	8.823	**5.651**	1.802	17.718	**0.003**
HNA-5 genotype (*ITGAL*01*01*)	–0.134	0.336	0.159	0.875	0.453	1.690	0.690
Constant	–2.685	.684	15.410				

B, beta coefficient; BMI, body mass index; HNA, human neutrophil antigen; SE, standard error.

Note: This analysis included two dummy-coded variables for knee OA outcome: sex (1 = female, 0 = male), age (1 = ≥57-year-old, 0 = <57-year-old), BMI (1 = ≥25.0, 0 = <25.0), HNA-1 genotype (1 = *FCGR3B*01*01*, 0 = otherwise), HNA-3 genotype (1 = *SLC44A2*01*01*, 0 = otherwise), HNA-4 genotype (1 = *ITGAM*01*01*, 0 = *ITGAM*01*02*), and HNA-5 genotype (1 = *ITGAL*01*01*, 0 = otherwise).
